# Development of corticostriatal connectivity constrains goal-directed behavior during adolescence

**DOI:** 10.1038/s41467-017-01369-8

**Published:** 2017-11-28

**Authors:** Catherine Insel, Erik K. Kastman, Catherine R. Glenn, Leah H. Somerville

**Affiliations:** 1 Department of Psychology and Center for Brain Science, Harvard University, 52 Oxford Street, Room 290, Cambridge, MA 02138 USA; 2 Department of Clinical and Social Sciences in Psychology, University of Rochester, 460 Meliora Hall, Rochester, NY 14627 USA

## Abstract

When pursuing high-value goals, mature individuals typically titrate cognitive performance according to environmental demands. However, it remains unclear whether adolescents similarly integrate value-based goals to selectively enhance goal-directed behavior. We used a value-contingent cognitive control task during fMRI to assess how stakes—the value of a prospective outcome—modulate flexible goal-directed behavior and underlying neurocognitive processes. Here we demonstrate that while adults enhance performance during high stakes, adolescents perform similarly during low and high stakes conditions. The developmental emergence of value-contingent performance is mediated by connectivity between the striatum and prefrontal cortex; this connectivity selectively increases during high stakes and with age. These findings suggest that adolescents may not benefit from high stakes to the same degree adults do—a behavioral profile that may be constrained by ongoing maturation of corticostriatal connectivity. We propose that late development of corticostriatal connectivity sets the stage for optimal goal-directed behavior.

## Abstract (web summary)

Adults adjust their cognitive performance according to the value of the outcome, but it is unclear whether adolescents do too. Here, authors show that adolescents do not adjust their cognitive effort according to value, and that this ability is mediated by connectivity between the striatum and prefrontal cortex.

## Introduction

The ability to titrate one’s cognitive performance to environmental demands is a fundamental aspect of optimal goal-directed behavior^1^. Rather than maximizing cognitive effort at every moment, adults selectively deploy cognitive resources when the outcomes are worthwhile^1, 2^. For example, an individual is likely to invest additional time and effort to prepare for a major job review compared to a routine meeting with her boss. Past work has shown that the value of prospective outcomes within a given context, which we will refer to as stakes, guide the computations that selectively titrate cognitive control^3–6^.

Contemporary neural and computational models of motivation–cognition interactions posit that value-cues shape motor selection and action via interactions among the ventral striatum (VS), dorsal striatum, and prefrontal cortex^1, 7–10^. Prefrontal cortical systems support effortful cognitive control processes, including context monitoring and action selection.^11–14^ These prefrontal systems are interconnected with striatal regions that code the motivational value of prospective incentives and guide optimal actions, which, in turn, maximize future outcomes^15–17^. Computational network models propose that the striatum serves a gating function^8, 18^, orchestrating goal-directed titration of cognitive and motor control. Dopamine-mediated value signals in the VS project to the dorsal striatum via indirect, looped connections^9, 19^. Accordingly, the striatum is thought to modulate the active maintenance of goal states in the prefrontal cortex^20^ and motor action selection via output gating. This selective gating determines how goal states influence appropriate action decisions in a context-dependent manner (i.e., selecting the appropriate action in response to a given stimulus)^20^. As such, the prospective value of an action can influence its selection and execution in the moment. Consistent with this model, adults improve performance in high stakes contexts^21–24^. Further, enhanced high stakes performance is paralleled by upregulated functional recruitment of prefrontal systems^1, 25, 26^ and increased corticostriatal connectivity^24, 27^.

Theoretical frameworks of adolescent neurodevelopment emphasize the ongoing maturation of corticostriatal circuit function^28, 29^. However, it remains unclear whether developmental changes in corticostriatal recruitment and connectivity enable optimal integration of value and cognitive control signals. While prior work has broadly implicated this circuitry in the maturation of cognitive control, reinforcement learning, and value-based decision making^30–33^, more evidence is needed to establish key links between age-dependent changes in brain function and developmental changes in behavioral performance during incentivized cognitive control.

While selective resource allocation is optimal for goal-directed behavior, research has not yet identified when, over the course of development, individuals gain the capacity to selectively titrate cognitive performance according to contextual demands. Although prior developmental work has examined the effects of incentives on cognitive performance, the findings have been largely inconsistent. Some studies suggest that incentives facilitate adolescents’ cognitive control^34–37^, whereas others have found no effect of incentives on adolescent performance^38, 39^. Yet other studies show no effect of incentives on adults’ cognitive control^40^, which conflicts with the literature on adult value-contingent performance^21, 23, 24, 41, 42^. Further, it remains unknown how high stakes influence adolescents’ cognitive control in maximally reactive control contexts^43^ (such as the go/no-go task context) where the need for control is not signaled in advance and can only be instantiated in the moment, which requires flexible action selection and continual context monitoring^14, 44^.

Here, we use a novel task and integrative analysis framework to identify the key components of corticostriatal neurodevelopment that mediate the capacity to improve goal-directed behavior when stakes are high. Participants ranging in age from 13 to 20 years completed a task during functional magnetic resonance imaging (fMRI) that required flexible action selection and allocation of cognitive control under high and low financial stakes, with high stakes defined as the relatively higher-valued prospective outcome. Participants first viewed a stakes cue, signaling a low or high stakes block. In high stakes conditions, correct responses were rewarded $1 and errors incurred a loss of $0.50; whereas for low stakes, correct responses were rewarded $0.20 and errors incurred a loss of $0.10. Next, participants completed a go/no-go task, followed by performance-contingent feedback at the end of the block.

Analyses addressed (i) the distinct effects of stakes on flexible cognitive performance from adolescence through early adulthood, and (ii) the neurodevelopmental changes in brain function that mediate the distinct effects of stakes on behavioral performance across development. Based on prior work demonstrating that adolescence is a developmental period of enhanced reward-seeking behavior^45^, it is plausible that adolescents would boost performance in high stakes conditions. On the other hand, corticostriatal networks continue to develop through adolescence^28, 29^, which could alternatively limit the degree to which adolescents titrate cognitive control according to contextual signals of value.

Here, we report results that are consistent with the latter possibility, although older participants selectively improve performance during high stakes, younger participants did not show evidence of improvement. This age-dependent behavioral phenomenon is mediated by emerging corticostriatal connectivity, which both increases with age and predicts stakes-based performance enhancement. These findings inform basic theory regarding the mechanisms of motivation–cognition interactions, developmental theory regarding the emergence of goal-directed behavior during adolescence, and have practical implications for adolescents’ capacity to selectively capitalize on high stakes situations in everyday life.

## Results

### Stakes-based performance improvements increased with age

**Fig. 1:**
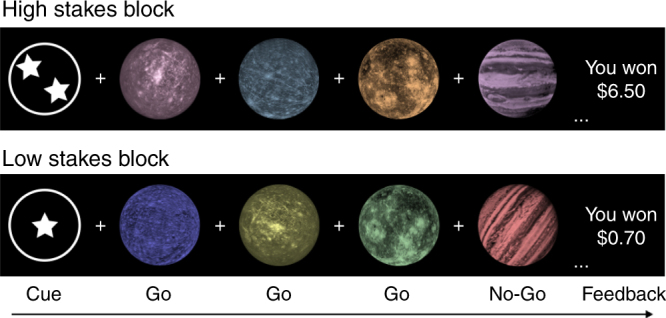
Planets task. Participants viewed a low stakes or high stakes cue followed by a series of eight target trials. In this example, participants were instructed to press a button for go trials (planets with craters) and withhold a response for no-go trials (planets with stripes). Correct responses were rewarded with $1.00 in high stakes and $0.20 in low stakes. Incorrect responses incurred a loss of $0.50 for high stakes and $0.10 for low stakes. Performance-contingent feedback was displayed at the end of a block

**Fig. 2:**
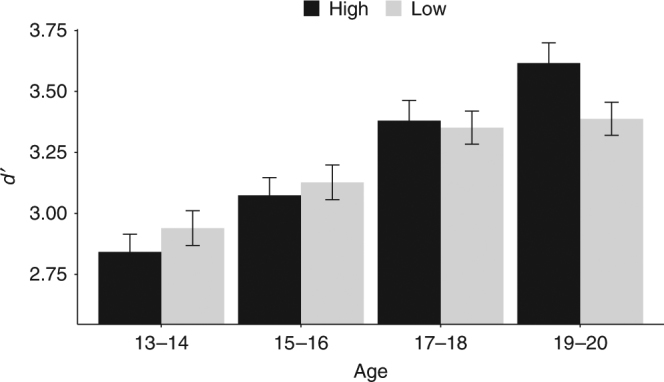
Performance by age. There is a significant interaction between stakes and continuous age, with an emerging rise in performance under high compared to low stakes with increasing age (*F*_(1,86)_ = 4.24, *p* = 0.04). The *x*-axis depicts age in two-year bins (*n* = 88 total, grouped for visualization purposes). D′ (*y*-axis) represents cognitive control performance. High (black bars) and low (gray bars) denote trial stakes. Bar heights represent the mean and error bars denote ±1 standard error of the mean. For individual data, see Supplementary Fig. 2

Behavioral analyses assessed the effects of stakes and age on task performance (Fig. 1), as measured by dprime (*d*′), a conglomerate measure of the capacity to discriminate, select, and execute the correct behavioral response (pressing to go trials and not pressing to no-go trials). Results revealed an expected main effect of age on *d*′ (*F*_(1,86)_ = 8.08, *p* = 0.006), with overall accuracy improving with age. The age effect was qualified by a significant interaction between age and stakes (*F*_(1,86)_ = 4.24, *p* = 0.04; Fig. 2). With age, there was an emerging tendency to selectively improve performance for high stakes conditions. The main effect of stakes was not significant (*F*(_1,86)_ = 0.16, *p* = 0.69). See Supplementary Table 3 for descriptive performance data and Supplementary Fig. 2 for individual data points.

To further examine the effects of age on high versus. low stakes *d*′ performance, post hoc tests were conducted for age bins of 2 years (ages 13–14, 15–16, 17–18, 19–20) using Tukey’s adjusted comparisons. These analyses revealed that only 19–20-year-olds exhibited significantly improved performance for high stakes (ages 13–14 (*n* = 20): *p* = 0.37; ages 15–16 (*n* = 26): *p* = 0.58; ages 17–18 (*n* = 22): *p* = 0.79; ages 19–20 (*n* = 20): *p* = 0.04). These findings suggest that value-based upregulation of cognitive control is developmentally constrained through adolescence.

While the go/no-go task is often used to isolate inhibitory control processes^46^, recent work has demonstrated that both go and no-go trials evoke shared control processes (e.g., momentary action selection and execution)^7, 14^. Thus, additional post hoc analyses were conducted to test whether stakes-based performance differences with age are specific to inhibitory motor processes (i.e., no-go stopping) or reflective of control processes common to both go and no-go trials. The stakes by age by target type interaction was not significant, *F*(_1__,__86_) = 0.05, *p* = 0.83, indicating that high stakes sharpen adult performance equivalently for go and no-go trials. More broadly, this finding suggests that high stakes facilitate not only motor inhibition per se but rather the superordinate process of flexible action selection and cognitive control.

Further analyses were conducted to evaluate the possibility that developmental differences in reaction time or speed/accuracy tradeoffs were driving the reported behavioral effects. Importantly, the stakes by age interaction remained significant when controlling for reaction time and stakes-related speeding (*F*(1,82) = 4.19, *p* = 0.04). Age was not associated with reaction time, and speed-accuracy trade-offs were not modulated by stakes (see Supplementary Note 2, Supplementary Tables 3 and 4 for full results). This suggests that the age by stakes interaction in performance did not result from developmental differences in speeding strategy or stakes-based changes in reaction time, consistent with the notion that development is placing constraints on performance rather than altering behavioral strategy.

### Corticostriatal activity increased for high stakes control

**Fig. 3:**
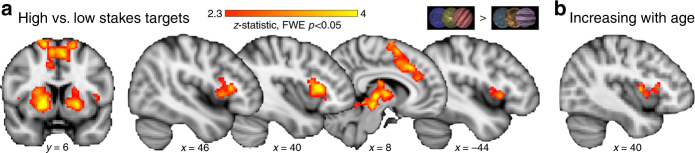
Stakes-selective functional activity. **a** High stakes versus low stakes targets activity. Whole-brain corrected statistical map illustrating regions exhibiting enhanced functional recruitment for high stakes relative to low stakes targets across all participants. Contrast represents whole-brain corrected *t* test (FWE *p* < 0.05). **b** Age covariate map. A covariate for age was added to the high > low targets contrast. Increasing age was associated with increased recruitment of bilateral vlPFC for high relative to low targets (right vlPFC shown, small volume corrected *t* test FWE *p* < 0.05)

Because the effect of high stakes improved go and no-go performance to an equivalent degree, go and no-go trials are considered jointly in neuroimaging analyses as an index of flexible action selection and execution of goal-directed behavior. Whole-brain contrasts revealed differential functional recruitment during the execution of control (Fig. 1; planet stimuli) for high stakes versus low stakes targets (combined go and no-go trials). This map identified activity in many nodes within the corticostriatal circuit, including significantly greater recruitment of the bilateral VS, dorsal striatum, ventrolateral prefrontal cortex (vlPFC), thalamus, and dorsal anterior cingulate/supplementary motor area for high stakes compared to low stakes trials (family-wise error (FWE) *p* < 0.05, Table 1, Fig. 3a). Separate contrasts for high stakes vs. low stakes go trials and high stakes versus low stakes no-go trials resulted in similar patterns of recruitment, further validating the combination of go and no-go trial types (Supplementary Fig. 3).

**Table 1:** fMRI activity for high versus low targets contrast

Region	*x*	*y*	*z*	*k*	*z*-stat
Thalamus	16	−10	4	981	4.72
Putamen	20	12	0		4.72
Pallidum	18	4	−2		4.30
Thalamus	−16	−12	6		4.08
Putamen	−20	8	0		4.07
Brainstem	2	−30	0		3.90
Pallidum	−12	2	0		3.77
Inferior frontal gyrus	42	20	10	152	3.66
Insula	30	18	10		3.54
Paracingulate gyrus	10	24	38	55	4.14
Frontal operculum cortex	−36	18	8	148	4.14
Superior frontal gyrus	16	4	68	34	4.02
Supplementary motor cortex	−4	4	56	112	4.02
Superior frontal gyrus	−12	4	66		3.91
Supplementary motor cortex	8	8	58	10	3.97

High > low stakes targets contrast, whole-brain FWE *p* < 0.05

To assess whether high versus low stakes functional recruitment was associated with age, a covariate for continuous linear increasing age was included as a predictor in the high stakes versus low stakes targets contrast. Increasing age was associated with enhanced functional recruitment of bilateral vlPFC (405 voxels at *x* = −32, *y* = 2, *z* = 6; 297 voxels at *x* = 36, *y* = 4, *z* = 8; SVC FWE *p* < 0.05, Fig. 3b), with high stakes upregulation of prefrontal recruitment increasing across adolescence.

To assess whether age-related changes in functional recruitment for high versus low stakes was associated with stakes-based performance differences, a covariate for high stakes *d*′ minus low stakes *d*′ (*d*′_high–low_) was included as a predictor in the high stakes versus low stakes targets contrast. No activity was observed within the vlPFC (*p* < 0.05 SVC FWE), and an exploratory whole-brain search also yielded no significant associations with *d*′_high–low_ at FWE *p* < 0.05. Thus, age-dependent changes in prefrontal cortical activity cannot account for differences in high stakes behavioral performance, motivating analyses focused on functional connectivity.

### High stakes corticostriatal connectivity emerges with age

We conducted psychophysiological interaction (PPI) analyses to examine how stakes modulate functional coupling with the striatum to specifically identify where there was enhanced connectivity during high stakes (high stakes versus low stakes targets contrast). Left and right VS were used as seed regions, given the VS’s role in valuation and motivated action (Methods). For analyses independent of age, high stakes elicited enhanced connectivity between the right VS and the parietal cortex (whole-brain FWE *p* < 0.05, 439 voxels at *x* = −44, *y* = −44, *z* = 38); no clusters survived correction for the left VS seed.

**Fig. 4:**
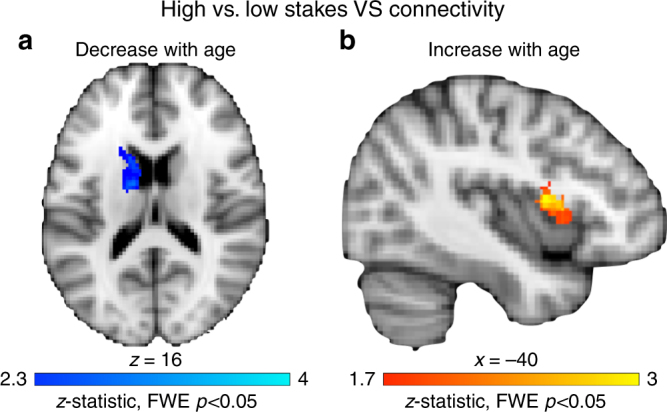
PPI for high stakes versus low stakes targets as a function of decreasing and increasing age. **a** Striato–striato coupling decreases with age. PPI results for high stakes versus low stakes targets with a ventral striatum seed that decreases with age (whole-brain corrected *t* test, FWE *p* < 0.05). Ventral striatum–caudate coupling was enhanced for high stakes in the youngest participants and decreased linearly across development. **b** Corticostriatal coupling increases with age. PPI results for high stakes versus low stakes targets with a ventral striatum seed that increases with age (small volume corrected *t* test, FWE *p* < 0.05). Ventral striatum–vlPFC connectivity increased for high stakes as age increased

The primary question, however, was to evaluate how stakes-selective striatal connectivity changed with age. Age was included as a whole-brain predictor in the PPI analysis for high stakes versus low stakes targets with the VS seeds. Results indicated significant high > low stakes coupling between the left VS seed and a cluster in left caudate (that extended into the putamen) that decreased with age (whole-brain *t* test, FWE *p* < 0.05, 334 voxels at *x* = −20, *y* = 20, *z* = 4, Fig. 4a). Thus, increased striato–striato coupling for high stakes was strongest in the youngest adolescents and decreased across adolescence. As age increased, there was enhanced high > low stakes coupling between the right VS and left vlPFC (SVC *t* test FWE *p* < 0.05, 215 voxels at *x* = −40, *y* = 4, *z* = 10, Fig. 4b). In sum, younger participants exhibited enhanced within-striatum connectivity for high stakes that decreased with age, whereas enhanced high stakes corticostriatal connectivity strengthened across adolescence. Thus, high stakes enhancements in connectivity shift from local striato–striato connections to long-range corticostriatal connections as individuals mature through adolescence.

### Corticostriatal connectivity and improved high stakes control

**Fig. 5:**
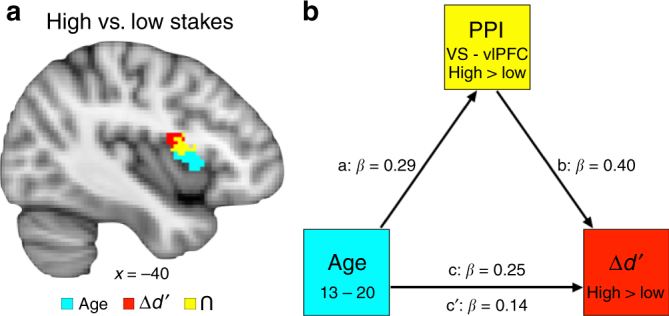
Stakes-selective functional connectivity. **a** Conjunction for high stakes versus low stakes targets PPI analysis with ventral striatum seed. PPI analyses were seeded in the ventral striatum to identify connectivity that was enhanced for high stakes relative to low stakes targets, and covariates were included for age (blue) and *d*′_high–low_ (red); (small volume corrected *t* tests, FWE *p* < 0.05). A conjunction analysis identified regions of overlap (yellow). **b** Ventral striatum–vlPFC connectivity mediation model. VS–vlPFC connectivity for high stakes versus low stakes targets mediated the relationship between age and improved performance for high versus low stakes conditions

To determine whether high stakes-selective connectivity was related to improved high stakes performance, *d*′_high–low_ was included as a predictor in the PPI analysis for high stakes versus low stakes targets with the VS seed. For the left and right VS seeds, *d*′_high–low_ was associated with enhanced coupling for high stakes in bilateral vlPFC (Fig. 5a; SVC *t* test, FWE *p* < 0.05, left seed: 222 voxels at *x* = −44, *y* = 8, *z* = −2; 319 voxels at *x* = 36, *y* = 12, *z* = 4; right seed: 291 voxels at *x* = −38, *y* = 4, *z* = 14; 422 voxels at *x* = 34, *y* = 12, *z* = 6). Therefore, individuals who showed a larger boost in high stakes performance also exhibited increased corticostriatal connectivity for high relative to low stakes trials.

### Connectivity mediated age and stakes-selective *d*′ association

A highly overlapping region of the left VLPFC exhibited enhanced stakes-dependent connectivity that was related both to age and improved performance (Fig. 5a). This permitted a formal mediation analysis to assess whether VS–vlPFC connectivity accounted for the relationship between greater age and greater boosts in performance for high versus low stakes. First, a conjunction analysis was computed between the PPI maps with predictors for age and *d*′_high–low_ (Fig. 5a), identifying a common region in the left vlPFC (215 voxels at *x* = −36, *y* = 10, *z* = 0). Parameter estimates for this region were extracted from the high stakes versus low stakes targets PPI, which represents the degree of differential VS–vlPFC coupling for high versus low stakes. The combined indirect mediation effect of the VS–vlPFC PPI connectivity was significant (indirect effect = 0.02, *p* = 0.006, CI [0.004, 0.05]), rendering the direct behavioral effect of age on *d*′_high–low_ nonsignificant (*c*: *β* = 0.25, *p* = 0.04 → *c*′: *β* = 0.14, *p* = 0.21; Fig. 5b). The indirect VS–vlPFC connectivity mediator accounted for 41.8% of the direct effect between age and *d*′_high–low_. This mediation effect suggests that high stakes upregulation of VS–vlPFC connectivity emerges through adolescence and accounts for the late emergence of stakes-based improvement in goal-directed behavior.

To test the robustness of the VS–vlPFC PPI mediator, additional analyses were conducted to rule out potential intervening behavioral or neurodevelopmental confounds. To do so, we added covariates to control for demographic differences (sex, estimated IQ), behavioral differences (RT_high–low_, task stimuli ratings), functional activity during task (VS and vlPFC), gray matter volumes (VS and vlPFC), and cortical thickness (vlPFC) (see Supplemental Note 4 for structural imaging analysis details). The mediation remained significant (indirect effect = 0.03, *p* = 0.02, CI[0.003, 0.06]), rendering the direct behavioral effect of age on *d*′_high–low_ nonsignificant (*c*: *β* = 0.35 *p* = 0.03 → *c*′: *β* = 0.21 *p* = 0.17)_._ By confirming that this effect cannot be explained by other relevant demographic, behavioral, or functional-neurodevelopmental differences, these control analyses demonstrate the high degree of selectivity of the VS–vlPFC connectivity mediation on stakes-based performance improvements.

### Stakes and incentive valuation were consistent across age

Analyses of subjective rating data were used to test the possibility that adolescents and adults valued the high and low stakes cues and outcomes differently than adults. See Supplementary Note 3 for full results. The data indicated that participants, regardless of age, assigned more positive valence and more arousal to the high stakes cues and outcomes relative to low stakes. Further, the relative difference in valuation ratings for high versus low stakes cues and outcomes was equivalent across the age range. Additionally, subjective value ratings did not influence task performance. These results build confidence that the observed developmental findings are not driven by differential motivation elicited by high versus low stakes.

### High stakes cue corticostriatal activity not related to age

**Fig. 6:**
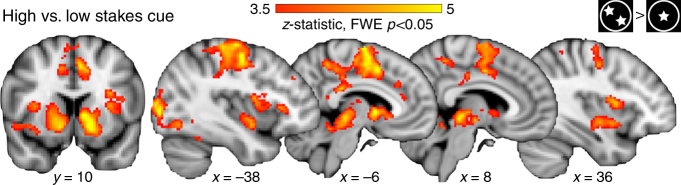
High stakes versus low stakes cue activity. Statistical map illustrating regions exhibiting greater functional recruitment for high stakes relative to low stakes cues across all participants. Stakes cue recruitment was not associated with age. Contrast represent whole-brain corrected *t* tests (FWE *p* < 0.05)

Another alternative explanation for the findings is that neural responses to the stakes cues varied with age, and that these differences may have driven the developmental shifts in motivated performance. For example, if adults upregulated corticostriatal preparatory–anticipatory recruitment while viewing high stakes cues predicting an upcoming block of high stakes trials, this might subsequently facilitate improvement on high stakes performance. Standard contrast analyses (whole-brain *t* tests) were performed on the neuroimaging data to test whether brain responses to the high versus low stakes cues changed with age. Across participants, the high stakes versus low stakes cues contrast revealed recruitment of the bilateral ventral and dorsal striatum, left precentral gyrus, right medial frontal gyrus, and occipital lobe (Fig. 6, Supplementary Table 6). Taken together, these results show that high stakes cues elicited enhanced corticostriatal activity during task preparation.

Age was added as a predictor to the high stakes versus low stakes cue contrast to assess whether enhanced high stakes cue activity varied with age. Notably, no regions showed significantly different recruitment with age, demonstrating that all ages exhibited equivalent increases in corticostriatal recruitment while preparing for a forthcoming block of high stakes relative to low stakes trials. Therefore, the developmental emergence of high stakes improvements cannot be explained by differential corticostriatal engagement during task preparation. Moreover, to the extent that ventral striatal responding could be considered an indirect metric of value anticipation^47, 48^, the consistency of responding across age provides further evidence that baseline valuation processes are comparable across the age range.

## Discussion

A key feature of goal-directed behavior is the capacity to selectively recruit cognitive resources in high stakes situations. In this study, we demonstrate (i) how the ability to capitalize on stakes to enhance cognitive control develops across adolescence, and (ii) reveal the neurocognitive mechanisms subserving the capacity to use stakes to selectively improve performance. Results demonstrated that adults enhanced the selection and execution of cognitive control during high stakes, consistent with prior research in adult samples^1, 21, 23, 24, 42^. However, throughout adolescence, performance for high and low stakes did not differ. These findings demonstrate that selectively titrating cognitive control performance according to changing motivational demands increases across adolescence. This developmental effect was mediated by functional connectivity between the VS and vlPFC. Specifically, emerging connectivity for high versus low stakes mediated age effects on stakes-based improvements in task performance. Together, these findings suggest that late maturation of corticostriatal connectivity supports developmental change in value-guided, goal-directed behavior.

Consistent with prior work in adults^1, 8^, we found that high stakes incentives improved adult performance during cognitive control. For adults, the ability to use stakes to guide performance was reflected in behavioral and neural mechanisms common to both going (go trials) and stopping (no-go trials) processes. Thus, adults do not use stakes to merely improve motor inhibition, they selectively improve reactive cognitive control processes that enable the selection and execution of the correct action at the correct time. This effect is consistent with the theoretical view that although no-go trials do require motoric stopping and can be used to isolate motor inhibition processes, there are also superordinate control processes commonly evoked by go and no-go trials, namely action selection, execution, and context monitoring^12, 14^. That being said, although the performance improvements observed in the adult group during the high stakes trials replicate patterns observed in prior studies of motivated cognition in adults^1, 8^, the present sample is limited in that the oldest participants in this study were 20 years of age. Given that the onset of adulthood is difficult to define^49^, and adolescence is a lengthy developmental phase, it is possible that the present adult group does not represent the final stage of cognitive maturation. Future work should test a wider age range to identify how stakes-based performance improvements stabilize over time.

Connectivity between the VS and vlPFC mediated improved performance for high stakes in adults, revealing a neural mechanism that can explain how stakes can be used to selectively titrate performance. Neural models of control underscore that the integration of signal in corticostriatal circuitry subserves the maintenance of goal states and regulation of action selection^1, 8^, with the VS and vlPFC serving as key nodes embedded within a distributed, highly interconnected circuit^50^. However, these regions do not share direct anatomical projections, suggesting that signals representing value and control demands integrate via indirect pathways enabled by the parallel, looped organization of corticostriatal circuitry^50^. In addition, high stakes cues, which signaled the stakes value of upcoming trials, heightened anticipatory striatal value signals. Notably, both adolescents and adults increased striatal activity for high stakes cues, suggesting that adolescents and adults processed high stakes contexts during the cue period equivalently. However, only older adolescents integrated this value representation during the task to successfully coordinate corticostriatal circuitry to guide subsequent performance in a stakes-based fashion.

Behavioral results revealed that younger adolescents aged 13–18 did not titrate cognitive control performance according to motivational context. This effect was unrelated to developmental differences in reaction time, which are often observed during cognitive performance in developmental samples. Additional analyses explored whether differential motivational processes may explain why adolescents did not improve for high stakes. One possibility could be that the adolescents were simply less motivated by the incentives. However, participants of all ages rated the high stakes cues and high monetary outcomes as equivalently more positive and more salient than low stakes cues. These subjective value analyses build confidence that valuation differences cannot explain the late development of stakes-based performance titration. Further, these findings suggest that the integration of valuation and cognitive control processes, rather than valuation processes alone, are most likely the cognitive mechanism underlying developmental differences in stakes-based performance improvements.

Connectivity analyses revealed that high stakes within-striatum connectivity (VS–caudate) decreased across adolescence, while high stakes corticostriatal connectivity (VS–vlPFC) increased with age (up until the age of 20). Computational models of motivated cognitive control suggest that value signals in the VS propagate to the dorsal striatum via spiraling connections with the midbrain^9^. Our findings reveal that adolescents exhibit value-based modulation of striatostriatal connectivity, whereas selective corticostriatal connectivity emerges later in development, suggesting that high stakes enhancements in connectivity shift from short-range to long-range as individuals mature into adulthood. This capacity to selectively upregulate corticostriatal connectivity for high stakes trials emerged in late adolescence, mediating improvement during high stakes conditions. These findings imply that selectively integrating striatal signals with prefrontal signals via stakes-specific connectivity could be a late-developing feature of corticostriatal maturation, which could constrain the titration of goal-directed behavior with value.

Our findings expand our understanding of the development of incentivized control in several ways. First, we found that adults improved performance under high stakes but adolescents did not. This result reflects a departure from prior developmental work suggesting that adolescents, but not adults, use rewards to improve cognitive control^36, 37^. However, this pattern has not been observed consistently in the literature^38, 39^ and these prior findings do not comport with studies of adults, which demonstrate flexible improvement in performance in high-value situations^21, 23, 24, 42^. The results of the present study thus provide a novel perspective on how stakes influence developmental changes in cognitive control. Specifically, when control must be implemented moment-to-moment in a flexible and reactive fashion, the ability to modulate behavior according to value-based goals may not emerge until late adolescence. Though these findings await replication, they align with theoretical accounts of motivated control based in adults^1, 8^ and provide new mechanistic targets for future research.

Second, the current study applies an analytical framework that directly links neurodevelopmental patterns with behavioral changes in performance. While prior work reports developmental differences in brain activity that are observed in parallel to behavioral differences^31, 36, 37^, here we expand on this work by demonstrating how specific neurodevelopmental processes guide age-related stakes-based performance improvements. More precisely, we describe specific corticostriatal connectivity mechanisms that guide successful motivation–cognition interactions. This connectivity mediation effect remains significant even while controlling for functional activity levels, structural (gray matter) development, and other potential confounds that also vary with age but fall short of explaining performance changes with age. Together, these advances permit inferences that link-specific patterns of adolescent neurodevelopment with developing changes in when and how incentives improve cognitive control. The selective titration of motivated cognition may evolve with age due to ongoing corticostriatal network reconfiguration, underscoring an emerging view that developmental changes in brain network coordination are especially important for behavioral outcomes^28^.

Taken together, the results presented here suggest that processes subserving the integration of value-cues with cognitive control demands—thereby selectively enhancing high stakes performance—continue to develop through adolescence. Future work should evaluate whether the mechanisms underlying such constraints are similar to what adults could face under different circumstances (e.g., if the stakes became much higher), or whether the mechanisms that subserve adolescents’ capacity constraints are developmentally unique. Prior work in adults has shown that while rewards can trigger improvements in adults’ cognitive control when the challenges are fairly easy, they fail to facilitate performance past a difficulty threshold^3^. It is plausible that adolescents have a lower threshold at which incentives stop helping cognitive control, and that the present study exceeded that threshold. It is also possible that adolescents and adults perform different cost/benefit calculations when deciding whether to engage in goal-directed cognitive effort. Future work would benefit from more objective measures of valuation and cognitive effort. The addition of experimental paradigms that measure willingness to work for high and low stakes could provide more direct evidence addressing the role of baseline valuation in motivation–control interactions across development. Moreover, future studies should include a neutral no-stakes condition to better characterize baseline control abilities, which would identify whether adults truly boost performance for high stakes or, conversely if they decrease performance for low stakes. Finally, future work should test a range of stakes-values to better identify how control performance scales with reward value to identify the point at which stakes help or harm control performance.

Finally, we believe that these findings could have implications for real world contexts in which adolescents could achieve their maximum cognitive potential. Adolescents are faced with mounting high stakes challenges as they approach adulthood, ranging from standardized testing to college applications. Prior work evaluating whether incentives and punishments facilitate cognitive performance in academic settings has been surprisingly mixed in its effectiveness^51, 52^. Our findings suggest that at least past a certain cognitive threshold, high stakes may not facilitate improvements in adolescents’ cognitive performance. More research is needed to specify the particular conditions under which high stakes conditions facilitate or impede cognitive performance across development.

## Methods

### Participants

Eighty-eight participants between the ages of 13 and 20 years took part in this experiment (42 females, Supplementary Fig. 1, Supplementary Note 1). Male and female participants represented in approximately equal proportions over the age range, verified with chi-squared analyses (Behavioral sample: *χ* ^2^ = 0.96, *p* = 0.81; MRI sample: *χ* ^2^ = 1.13, *p* = 0.77), and behavioral task performance was not influenced by participant sex (Supplementary Note 1). To ensure adequate sample size to test continuous age effects, we aimed to test ~10 participants per year of age, which was chosen based on sample sizes of previous work^31, 53.^ This sample size was determined prior to data collection based on sample sizes from recent studies assessing changes in cognitive control across age groups^31^ and developmental studies invoking age as a continuous predictor of changes within adolescence^53^. A power analysis could not be conducted because the primary manipulation of stakes on go/no-go performance has not been used in prior work to our knowledge.. Behavioral analyses included data from all participants. *N* = 16 participants were excluded from the fMRI sample due to excessive head motion (Supplementary Table 1), poor brain coverage, or incomplete scan. Participants were screened for past or current psychiatric or neurological illness and had no lifetime use of psychotropic medication. During study participation, participants completed the Similarities and Matrix Reasoning sections of the Wechsler Abbreviated Scale of Intelligence (WASI-II)^54^. Full-scale IQ was approximated using the age and sex specific *t*-score conversion. The sample was balanced on estimated IQ across the age range, as there was no significant association between IQ and age (*r*(75) = 0.10 *p* = 0.38; Supplementary Table 2). Before study participation, participants and their legal guardians provided written assent and consent under the protocol approved by the Committee for Use of Human Subjects at Harvard University. This study was conducted once and has not been replicated in our laboratory.

### Value-contingent cognitive control task

Participants performed the Planets Task during functional brain imaging (Fig. 1), which is an incentivized cognitive control task in which participants completed go/no-go blocks under high and low financial stakes. First, the participant viewed a cue indicating the stakes of the subsequent trials—low stakes (circle with one star) or high stakes (circle with two stars). Next, a series of eight targets were displayed, which included planets with craters (i.e., go targets), and planets with stripes (i.e., no-go targets). Participants were instructed to press the index finger of their dominant hand as quickly as possible when the go target appeared but to not press to no-go targets. Correct responses were rewarded at $0.20/trial for low stakes and $1.00/trial for high stakes. Incorrect responses incurred a loss of $0.10/trial for low stakes and $0.50 for high stakes. Following a series of eight targets, performance feedback displayed the participant’s cumulative earnings for the block. Participants were instructed before completing the task that a random selection of 20% of earnings would be paid out; however, all participants received $20 as bonus payment at the end of the study.

Target images consisted of planets with craters or stripes, which served as the go and no-go cues. Assignment of craters and stripes to the go or no-go conditions to low and high stakes was counterbalanced across participants. Square frames surrounding trial stimuli (one-line frame for low stakes and two-line frame for high stakes) differentiated task from rest and provided a constant reminder of stakes conditions to reduce working memory demands. The task was presented using Psychopy software version 1.79^55^ and displayed on a screen visible through a mirror attached to the head coil. Behavioral responses were collected with a MRI-compatible button box. FMRI BOLD activity was measured over four functional runs, lasting 3 min and 46 s (s) each.

Within a block of trials (Fig. 1), the cue indicating forthcoming high or low stakes was displayed for 1 s, the targets for 500 ms, and feedback indicating earnings for 1.5 s. Target stimuli were temporally separated by jittered interstimulus intervals ranging from 1.5 to 3.5 s (average 2.4 s). Between blocks, participants viewed a fixation crosshair for 10 s. The order of low and high stakes blocks was pseudo-randomized across runs, and run order was counterbalanced across participants.

Each run of the Planets Task (4 runs total) consisted of six intermixed blocks (three low stakes, three high stakes). In total, the task included 24 stakes cues, 192 targets (128 gos and 64 no-gos), and 24 feedback displays. The targets consisted of 66% go and 33% no-go trials. The prepotency of go trials preceding a no-go trial ranged from 0 (no-go following a no-go trial) to 4 (no-go following four go trials). The prepotency order was pseudo-randomized across blocks within each run and was equivalent for low and high stakes. The target images were randomly rotated in 45° intervals and were displayed in eight different colors to increase visual interest and mitigate habituation.

### Post-task ratings

Participants provided ratings of the stimuli and outcomes they experienced during the task. Participants used the Self-Assessment Manikin^56^ to rate the valence (positive to negative) and arousal (low to high) of the low and high stakes cues (one star or two stars) and the monetary amounts used in the task ($0.10, $0.20, $0.50, $1.00) (Supplementary Note 3).

### Image acquisition and quality assessment

Participants were scanned at the Center for Brain Science-Neuroimaging at Harvard University using a Siemens 3.0 T Tim Trio scanner with a 32-channel head coil. Anatomical data were acquired with a high-resolution, T1-weighted anatomical scan using a multiecho multiplanar rapidly acquired gradient-echo (MEMPRAGE) sequence (repetition time = 2530 ms, echo time = 1.74, 3.59, 5.44, 7.29 ms, flip angle = 7°, field of view = 212 mm, slice thickness = 1 mm, voxel size = 1 × 1 × 1 mm) that is robust to head motion^57^. Functional data were acquired with a T2*-weighted EPI sequence with the following parameters: repetition time = 2 s, echo time = 30 ms, field of view = 216 mm, flip angle = 90°, voxel size = 3 × 3 × 3 mm. Thirty-one slices aligned to the anterior to posterior commissure plane were acquired, with a slice thickness of 3.75 mm. Prospective acquisition correction for head motion was implemented during functional scans to adjust for head motion in real time and reduce motion-induced corruption of signal^58^.

Functional MRI data were carefully evaluated for motion and signal outliers given the negative impact it can have on signal quality and general linear model (GLM) estimates. The following rules were imposed for data exclusion. Runs in which more than 10% of TRs were censored for motion (relative motion > 1 mm, frame displacement calculated as average of rotation and translation parameter differences using the root mean square formulation, as implemented in FSL) or outlier signal intensity (exceeded the grand run median by 4.5 median absolute deviations) were excluded from analysis. Runs with a single-relative movement exceeding 5 mm were also excluded. Any participant with more than two excluded runs (out of four) was excluded completely from analysis. In total, seven participants had at least one run excluded (three with two excluded runs, four with one excluded run).

Analyses were conducted to rule out the possibility of age-related differences in the quantity of usable data across the sample. For the participants included in the fMRI group analyses, there was no significant relationship between total number of censored volumes and age (*r*(71) = 0.04, *p* = 0.74), censored volumes and overall performance (*r*(71) = −0.07, *p* = 0.56), or censored volumes and stakes-specific performance improvements (*r*(71) = −0.11, *p* = 0.38); Supplementary Table 5. In sum, participants regardless of age retained similar amounts of high quality data, and the quantity of data did not relate to the key behavioral measures of interest. These results build confidence that age-related changes in neural activity and connectivity cannot simply be attributed to differences in data quantity or quality.

### Analysis of behavioral data

Behavioral data analyses were conducted in R^59^. All *p*-values were computed for two-tailed tests. For go trials, a correct response was defined as making a button press within the 1.5 s response window. For no-go trials, a correct trial constituted withholding a response within the 1.5 s response window. D′ (*d*′), a conglomerate measure of the capacity to select and execute the correct behavioral response (pressing to go trials and not pressing to no-go trials), was calculated using standard methods (*d*′ = (*z*(hit rate)−*z*(false alarm rate)))^60^. Go and no-go accuracy was also assessed separately for effects of stakes and age (Supplementary Table 3).

To assess the effects of age, stakes, and age by stakes interactions, a repeated measures ANOVA was conducted with *d*′ performance as the outcome variable, stakes as a within-subjects factor (low, high), and a continuous predictor representing mean-centered age. Age was used as a continuous predictor of developmental differences to maximize statistical power and to mitigate the need to create semi-arbitrary boundaries between age groups^53^. Linear age differences were of primary interest given the age range, and the linear predictor was calculated by mean-centering each participant’s actual age. Post hoc comparisons were conducted to query stakes effects within 2-year age bins: 13–14 (*N* = 20), 15–16 (*N* = 26), 17–18 (*N* = 22), 19–20 (*N* = 20). Post hoc contrasts were conducted with the lsmeans package in R^61^, and adjusted *p*-values were computed using the Tukey’s method for multiple comparisons. Levene tests confirmed homogeneity of variance across groups.

Because cognitive development may follow nonlinear trajectories, we also evaluated two nonlinear age models: (1) a quadratic model to test whether performance peaked during mid-adolescence; (2) a cubic model to test whether performance increased during adolescence and stabilized into adulthood^53, 62^. To compute and compare linear, quadratic, and cubic age terms, the poly function was used in conjunction with the nlme package^63^ in R. The poly function computes orthogonal polynomials, which allows for inclusion of multiple age terms within the same model. Three random effects models were computed with the nlme package: (1) linear age only, (2) linear and quadratic age terms, (3) linear, quadratic, and cubic age terms. To assess model fit, the anova function in R was used to determine whether the added polynomial terms increased model fit. Neither the addition of the quadratic term (model 2), or of both the quadratic and cubic terms (model 3) improved model fit (AIC_linear_ = 342.11, AIC_quadratic_ = 344.52, AIC_cubic_ = 348.41). Further, neither quadratic nor cubic age was associated with performance, and neither term interacted with stakes. The linear age and the linear age by stakes interaction terms remained significant when these additional nonlinear terms were included in the model, confirming that a linear age trajectory best captured the observed developmental shift in high stakes performance. A separate age model was examined to test whether an inverse age model (calculated as 1/age then mean-centered) was predictive of performance differences^64, 65^. Model comparison revealed that the linear age predictor model provided a better fit than the inverse age predictor model, as indicated by lower AIC values (AIC_linear_ = 342.11, AIC_inverse_ = 342.32). Thus, the inverse model was not interrogated further.

Reaction time was measured for correct go trials to test for the possibility of age differences in speed-accuracy tradeoff mechanisms for high versus low stakes (see Supplementary Note 2 and Supplementary Table 4 for analyses ruling out speed-accuracy tradeoff confounds with age).

### Analysis of fMRI data

FMRI data analysis was performed in FSL (version 5.0.4)^66^. Preprocessing was conducted in FSL through the Lyman pipeline (v. 0.0.7), which is based on the Nipype project (v. 0.9.2)^67^. Analysis and pipeline code is available upon request. Preprocessing steps included slice-time correction, realignment, coregistration of functional to structural images using bbregister^68^, nonlinear normalization of structural to FSL’s MNI152 template space using ANTS (1.9.x, svn release 891)^69^, and spatial smoothing with a 6 mm Gaussian kernel. Stringent inclusion criteria were applied based on data quality, and data quality was not related to age or behavioral task performance (Supplementary Table 5).

Preprocessed BOLD data were submitted to a GLM analysis using film_gls in FSL to estimate relevant task effects. The model regressors included temporal onsets for (1) low stakes cue, (2) high stakes cue, (3) low stakes go, (4) low stakes no-go, (5) high stakes go, (6) high stakes no-go, (7) low stakes feedback, (8) high stakes feedback, (9) errors (incorrect go and no-go trials). Error trials were modeled as a separate regressor of non-interest. Results from the reported contrasts only include correct trials. Task regressors were convolved with the canonical hemodynamic response function. Nuisance regressors included 6-parameter motion correction values calculated using mcflirt (FSL), censored frames for deviant signal intensity, and censored frames for excessive motion.

Random effects group analyses were conducted with three goals: (a) to identify task-based changes in functional activity for high stakes versus low stakes targets, (b) to identify relationships between age and performance and high stakes versus low stakes functional activity, and (c) to identify relationships between age and performance and high stakes versus low stakes functional connectivity. These analyses consistently identified stakes-based modulation in the VS and vlPFC, consistent with a host of prior work^1, 8, 14, 70^, justifying these regions to be of primary interest for further analyses.

Analyses aimed to identify task activation patterns, described in (a) above, are thresholded using whole-brain correction of *z* > 2.3 using FLAME 1 + 2, as implemented in FSL, resulting in whole-brain threshold of *p* < 0.05 FWE corrected. The vlPFC was considered an a priori region of interest for subsequent analyses, given its central role in cognitive control processes, including in prior work using go/no-go tasks^71–73^. To define the specific localization of vlPFC response from this data set, we first identified bilateral vlPFC clusters from the independent functional contrast of high > low stakes cues, thresholded at whole-brain *p* < 0.05 FWE corrected. These vlPFC regions formed a restricted search space (i.e., small volume mask) within which we queried for relationships with age and with behavior (steps (b) and (c) above). Age-associated and behavior-associated maps were thresholded using FWE correction of *p* < 0.05 within the vlPFC masks (i.e., *p* < 0.05 FWE SVC), implemented using the easythresh function in FSL.

Random effects group analyses identified task-based changes in functional activity for high stakes versus low stakes targets. Go and no-go trials were combined at the contrast level because behavioral analyses revealed that stakes improved performance for go and no-go trials. Moreover, separate analyses of high versus low go and high versus low no-go contrasts (thresholded at whole-brain *p* < 0.05, FWE corrected) revealed highly overlapping patterns of recruitment (Supplementary Fig. 3). Task timings permitted independent analyses of the cue (high > low cue) used for control analyses (Results), and feedback (high > low feedback, Supplementary Fig. 4; Supplementary Table 7). Cluster tables were generated for the main contrasts based on whole-brain *p* < 0.05 FWE correction. Region labels are based on the Harvard-Oxford Cortical and Subcortical Atlases^74^. Subclusters were defined by local maxima (activation peaks) within each cluster using a higher-values-first watershed searching algorithm^75^, implemented in MATLAB^76^. If multiple subclusters were identified within the same anatomical region, the strongest activated coordinates in each hemisphere was retained and redundant weaker entries were omitted.

Contrasts of interest were constructed with an age covariate to identify developmental differences in task execution (high > low targets) and evaluated statistically using one-sample *t* tests thresholded at *p* < 0.05, FWE SVC. A similar analysis was conducted for stakes-based performance (*d*′_high–low_) to identify brain regions whose differential high versus low stakes activity correlated with improved performance for high versus low stakes trials.

PPI analyses were conducted to assess differential functional connectivity during high stakes versus low stakes targets^77, 78^. First, seed regions in the left and right VS were defined anatomically based on the maximum probabilistic mask from the Harvard-Oxford Subcortical Atlas in FSL^74^. The VS seed was selected to query how value-related signals modulate cortical engagement during cognitive control, based on a priori hypotheses regarding the role of the VS in valuation and motivated action^8–10^. Additionally, reward responses in the VS have been shown to undergo key functional changes during adolescence^62,79^. Analyses for the left and right VS seeds were conducted separately.

A pair of PPI specific general linear models (one per VS seed) was constructed with the raw seed timecourse data, the temporal onsets for the high versus low stakes targets, and the product of the timecourse and contrast onsets. PPI analyses incorporated additional timeseries regressors for mean white matter signal and ventricular signal into the General Linear Model^80^ to reduce potential noise confounds. White matter and ventricle masks were defined for each subject in Freesurfer space^81^ using the mri_binarize command, and then affine transformed into subject specific T1 space using the flirt command in FSL^66^. The extracted timeseries data for the separate ventricle and white matter masks were then added to the PPI general linear model as nuisance regressors. The six motion parameters for translations and rotations were also included as nuisance regressors to reduce motion-induced confounds which have been shown to distort connectivity profiles, especially in developmental samples^82, 83^. Fixed effects models were conducted at the subject level and then submitted to a random effects analysis to compute the group-level statistics focused on the psychophysiological interaction regressor.

Random effects analyses were conducted to identify age effects on connectivity using the mean-centered linear age regressor as a predictor of differential VS functional connectivity for high stakes versus low stakes targets. Two separate age models were computed. The primary age model implemented an age-increasing covariate to identify task-based connectivity that increased across development (threshold: *p* < 0.05, FWE SVC corrected for vlPFC, see above). The second exploratory age model implemented an age-decreasing covariate to identify task-based connectivity that decreased across development (threshold: whole-brain *p* < 0.05, FWE). Finally, a separate whole-brain random effects analysis was conducted to identify brain-behavioral linkages between VS functional connectivity for high > low targets and *d*′_high–low_. This model implemented covariate representing *d*′_high–low_ to identify task-based connectivity that increased with increasing stakes-based performance (threshold: *p* < 0.05, FWE SVC corrected for vlPFC).

### Mediation analysis

Identifying both age-related changes and brain-behavior linkages between VS and vlPFC permitted formal mediation testing. A mediation analysis was conducted to test whether the indirect effect of VS–vlPFC connectivity mediated the relationship between increasing age and increasing performance improvements for high stakes versus low stakes. To identify ROI clusters for the mediation analyses, a conjunction analysis was first conducted to find regions of overlap between the PPI map that including the age covariate and the map including the *d*′_high–low_ covariate. This conjunction isolated a cluster (105 voxels at *x* = −32, *y* = 6, *z* = 6) in the left vlPFC. Next, connectivity weights reflecting PPI strength for high stakes versus low stakes targets were extracted from this region for each participant.

A mediation model was computed to test the indirect effect of VS -vlPFC connectivity as a mediator of the relationship between age by stakes on *d*′ performance. The direct path (c path) consisted of the linear model testing the effects of age on high versus low *d*′ performance. The indirect path consisted of VS–vlPFC connectivity for high stakes versus low stakes targets. The a path comprised the linear model of age predicting VS–vlPFC connectivity, and the *b* path comprised the linear model of VS–vlPFC connectivity predicting high versus low *d*′ performance. A robust mediation analysis was computed with the WRS2 package in R^84^ which down-weights the influence of outliers. The ZYmediate function was used to compute the robust estimate of the mediating effect (indirect path)^84^. Nonparametric bootstrapping procedures estimated the confidence intervals and *p*-value using 1000 samples. The proportion mediated was quantified by determining the proportion of the indirect effect mediating the direct effect: (*a*×*b*)/(*c* + (*a*×*b*))^85^.

An additional mediation analysis was performed to evaluate whether the significant mediation result was robust to the influence of several potential confounding variables. To do so, we computed covariates representing (a) sex, (b) estimated IQ, (c) stakes-based reaction time changes (RT_high–low_), (d) valence and arousal ratings of high vs low stakes cues (cue_high–low_), (e) functional activity in the right VS and left vlPFC for the high > low cue and high > low targets contrasts, (f) structural gray matter volume and thickness within the PPI ROIs (right VS volume, left vlPFC volume, left vlPFC thickness). Note that thickness estimates are constrained to the cortical surface, hence there is no thickness measure for the VS (see Supplementary Note 4 for details and Supplementary Table 8 for structural analyses). Confounds were added as to evaluate whether the primary mediation finding remained significant when these continuous covariates were included in the model. The mediate function from the mediation package^85^ in R was implemented to compute this mediation with additional covariates. When these covariates were added to the model, the *a*, *b*, and *c* paths remained significant (*a* path: *β* = 0.34 *p* = 0.02, *b* path: *β* = 0.47 *p* = 0.001, *c* path *β* = 0.35 *p* = 0.03).

### Code availability

Neuroimaging analysis code was based on the Lyman pipeline (v. 0.0.7, available at https://github.com/mwaskom/lyman). Customized neuroimaging analysis code and behavioral task code are available at https://github.com/kinsel.

### Data availability

Behavioral data are available at https://github.com/kinsel. Unthresholded maps from the neuroimaging analyses are available at *Neurovault* (https://neurovault.org/collections/2698).

## Electronic supplementary material

Supplementary Information
