# Supplementary material for: Development of corticostriatal connectivity constrains goal-directed behavior during adolescence
Source: Nat Commun. 2017 Nov 28;8:1605. doi: 10.1038/s41467-017-01369-8 (PMC5705718; doi:10.1038/s41467-017-01369-8)
Supplement: Supplementary file 1 — Supplementary Information [file 41467_2017_1369_MOESM1_ESM.pdf]

Supplementary Note 1. Sex difference analyses.

To verify that participant sex did not influence the main results, we re-ran our primary behavioral analysis with sex as a covariate (d'~stakes x age x sex). There was no main effect of sex on overall d' performance (F(1,84)=2.37, p=0.12), the stakes by sex interaction was not significant (F(1,84)=0.007, p=0.93), and the stakes by age by sex interaction was also not significant (F(1,84)=2.41, p=0.12). Importantly, when controlling for sex, the stakes x age interaction remained significant (F(1,85)=4.26, p=0.042). Together, these results confirm that sex did not bias the reported results focused on high vs low stakes.

Supplementary Note 2. Reaction time analyses.

Reaction time analyses were conducted to test for the possibility that the stakes by age interaction in overall performance resulted from a stakes-specific developmental difference in reaction times and speed/accuracy tradeoffs. First, we assessed whether stakes and age influenced reaction time (RT~stakes x age). There was a trend-level effect of stakes on reaction time (F(1,86)=2.98, p=0.09), such that reaction times were marginally faster in high compared to low trials (M_{high}=488 ms, M_{low}=491 ms). The main effect of age was not significant (F(1,86)=0, p=0.99). The age by stakes interaction was also not significant (F(1,86)=0.04, p=0.84).

Subsequent analyses were conducted to assess speed accuracy tradeoffs. These analyses assessed whether the performance was modulated by reaction time, stakes, and age by computing a model with d' as the outcome variable and age, stakes, and reaction time, and the 2-way and 3-way interactions as predictors (d'~stakes x age x RT). Importantly, the stakes by age interaction reported in the main manuscript remained significant when controlling for reaction time in the same model (F(1,82)=4.19, p=0.04). There was a significant main effect of reaction time on overall performance accuracy (F(1,82)=8.17, p=0.005), such that faster reaction times were associated with worse performance across both low and high conditions. This main effect was qualified by a significant age by reaction time interaction (F(1,82)=6.28,

p=0.01), such that faster responses were associated with poorer overall performance in younger, but not older, participants. However, critically this relationship did not interact with stakes, as the age by reaction time by stakes interaction was not a significant predictor of d' performance (F(1,82)=1.47, p=0.23). That is, while younger participants were more susceptible to speed accuracy trade-offs, this occurred in both low and high stakes conditions equivalently.

Taken together, these analyses demonstrate that adolescents did not exhibit faster reaction times during high stakes trials, compared to low stakes, nor did they exhibit enhanced speed-accuracy tradeoffs in high stakes conditions. Therefore, adolescents' inability to upregulate performance for high stakes is not merely an artifact of stakes-specific speeding-related errors. These findings demonstrate that any differences of age on reaction time were global and did not affect the key stakes-dependent performance differences which were the focus of this paper.

Supplementary Note 3. Subjective value ratings.

To quantify the subjective valuation of task cues and monetary outcomes, participants rated task related stimuli using a 1-9 scale for valence and arousal. Ratings data were missing from 9 participants, so the following analyses include data from 81 participants out of the full sample of 88. Separate repeated measures ANOVAs were conducted for valence and arousal using rating as the outcome variable and stakes, age, and the interaction as predictors (rating~stakes x age). For the preparatory stakes cues (star stimuli indicating the stakes of upcoming trials), the main effect of stakes was significant for ratings of arousal (F(1,78)=72.92, p<0.001) and valence (F(1,78)=102.86, p<0.0001), suggesting that regardless of age, participants experienced the high stakes cues as significantly more positive and higher in arousal. The age by stakes interaction was not significant for arousal (F(1,78)=0.09, p=0.77) or valence (F(1,78)=2.18, p=0.14), indicating that there were no confounding effects between age and subject valuation of the task cues.

Participants also rated the valence and arousal of the win and loss amounts for high and low stakes (low +$0.20/-$0.10, high +$1.00/-$0.50). Repeated measures ANOVAs were computed for both valence and arousal with rating as the outcome variable and age and stakes as predictors (rating~stakes x age). Separate analyses were conducted for win and loss amounts. For the win amounts, there was a main effect of stakes for valence ($F(1,77)=37.16$, $p<0.001$) and arousal ($F(1,77)=89.19$, $p<0.001$) suggesting that all participants, regardless of age, experienced the high stakes wins amounts as significantly more positive and higher in arousal. The age by stakes interaction was not significant for valence ($F(1,77)=0.56$, $p=0.5$), or arousal ($F(1,77)=0.45$, $p=0.5$), indicating that there were no confounding effects between age and subject valuation of the monetary gains employed in the task.

For the loss amounts, there was a significant main effect of stakes for valence ($F(1,77)=35.87$, $p<0.001$) and arousal ($F(1,77)=63.75$, $p<0.001$) suggesting that all participants, regardless of age, experienced the high stakes loss amounts as significantly more negative and higher in arousal. The age by stakes interaction was not significant for valence ($F(1,77)=0.31$, $p=0.6$) or arousal ($F(1,77)=0.24$, $p=0.6$), indicating that there were no confounding effects between age and subject valuation of the monetary losses employed in the task.

Individual-differences analyses were conducted to assess whether cue ratings influenced stakes-based task performance. Specifically, we sought to evaluate whether individuals who rated the high stakes cue as significantly more positive and arousing than the low stakes cue exhibited relatively higher boosts in high stakes performance or speeded reaction times.

To test this, we computed difference scores for the stakes cue ratings (high cue – low cue ratings for $valence_{high\text{-}low}$ and $arousal_{high\text{-}low}$), d' performance ($d'_{high\text{-}low}$ = high d' – low d') and RT ($RT_{high\text{-}low}$ = high go RT – low go RT). Multiple linear regressions with age and rating and age as predictors were computed separately for $d'_{high\text{-}low}$ and $RT_{high\text{-}low}$ outcome variables. Because $valence_{high\text{-}low}$ and $arousal_{high\text{-}low}$ were highly correlated ($r(79)=0.5$, $p<0.0001$), $valence_{high\text{-}low}$ and

$arousal_{high-low}$ predictors were included in separate models. For $d'_{high-low}$, there was no influence of either $valence_{high-low}$ (b=0.23, p=0.49) or $arousal_{high-low}$ (b=0.21, p=0.47) on stakes-related performance differences. For $RT_{high-low}$, there was also no effect of $valence_{high-low}$ (b=0.0003, p=0.82) or $arousal_{high-low}$ (b=0.006, p=0.62) on speeding for high compared to low stakes. Together, these results confirm that subjective valuation of the stakes cues did not influence stakes-based performance differences.

Supplementary Note 4. Structural region of interest analyses.

Structural analyses were conducted to query for a confounding influence of gray matter development of the prefrontal cortex and striatum on functional connectivity. Structural analyses were implemented with Freesurfer[1] 5.3.1 standard recon-all processing streams to compute estimates of grey matter thickness and cortical volume for each participant for cortical regions, and grey matter volume only for subcortical regions. Multiple structural measures were calculated for the vlPFC because recent developmental work has shown that cortical thinning, which can be characterized by cortical thickness measures, may influence developmental changes in cortical volume[2]. Regions of interest for the vlPFC and striatum were extracted from the Desikan-Killiany 2005 atlas[3] according to the automatic parcellation. For the vlPFC, thickness and volume were calculated for the left and right Pars opercularis and Pars triangularis. For the striatum, volume was calculated for the left and right caudate, putamen, and nucleus accumbens. Pearson correlations were computed to assess relationships of vlPFC structure (thickness and volume) and striatal volume with age, d', and $d'_{high-low}$. Results are listed in Supplementary Table 8.

Right nucleus accumbens volume and left Pars opercularis volume and thickness were added as covariates to the mediation model, given the overlap with the right VS seed and left vlPFC cluster from the functional connectivity analysis. While we did observe expected age-

related reductions in cortical thickness, including these estimates as covariates did not influence the significance of the connectivity mediation.

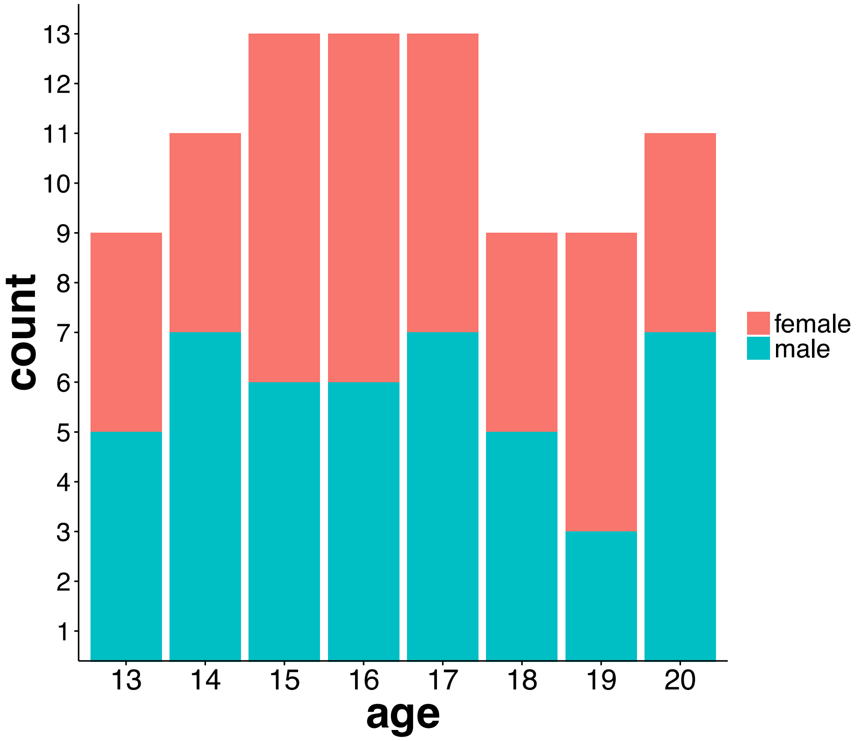

Supplementary Figure 1. Sample N=88 characterized by age and sex. 13 year olds, 5 male, 4 female; 14 year olds, 7 male, 4 female; 15 year olds, 6 male, 7 female; 16 year olds, 6 male, 7 female; 17 year olds, 7 male, 6 female; 18 year olds, 5 male, 4 female; 19 year olds, 3 male, 6 female; 20 year olds, 7 male, 4 female.

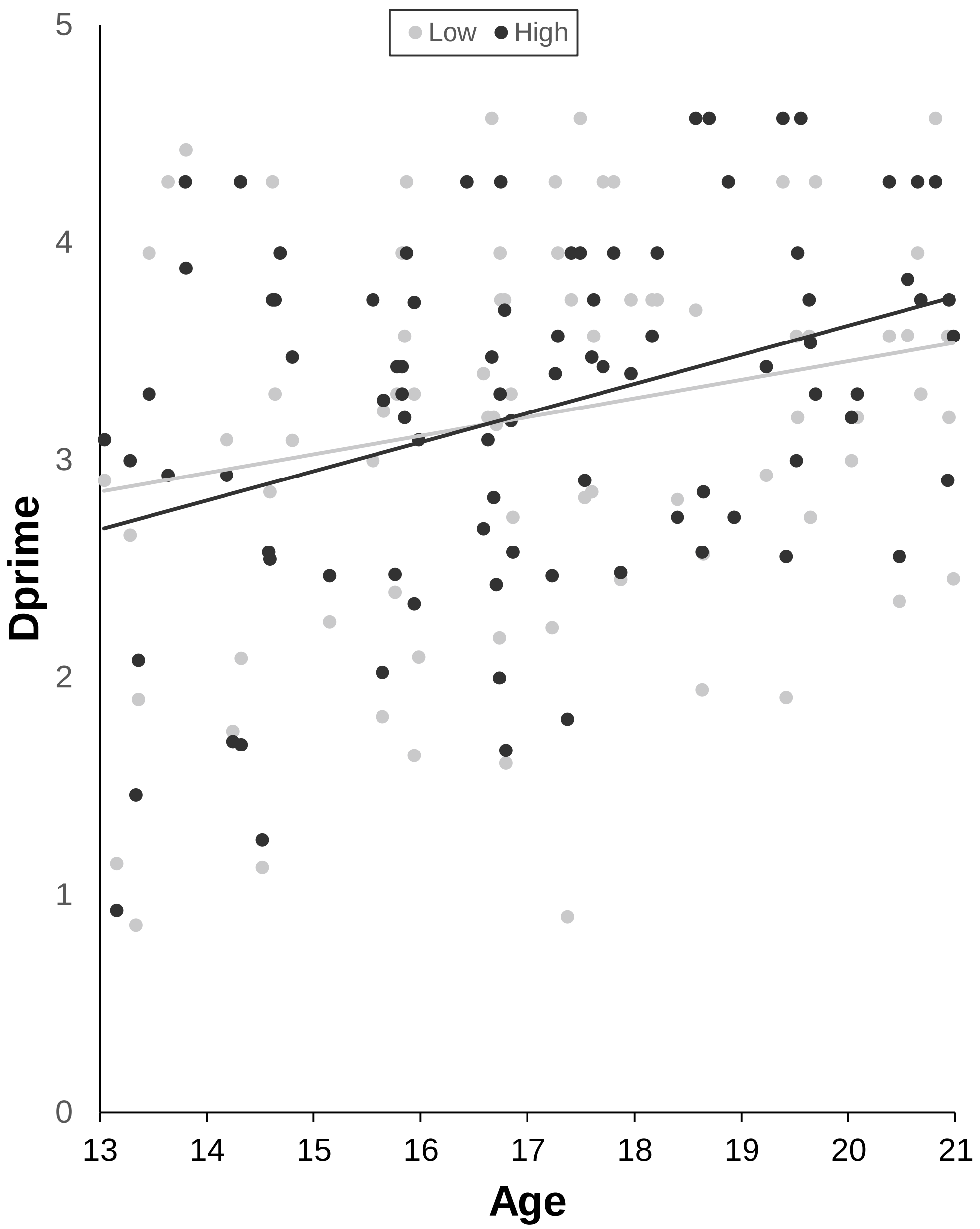

Supplementary Figure 2. Performance by continuous age. There is a significant interaction between stakes and continuous age, with an emerging rise in performance under high compared to low stakes with increasing age ($F(1,86)=4.24$, $p=0.04$). The x-axis depicts continous age (n=88 total). Dprime (y-axis) represents cognitive control performance. High (black points) and low (gray points) denote trial stakes. Lines represent the the regression fit.

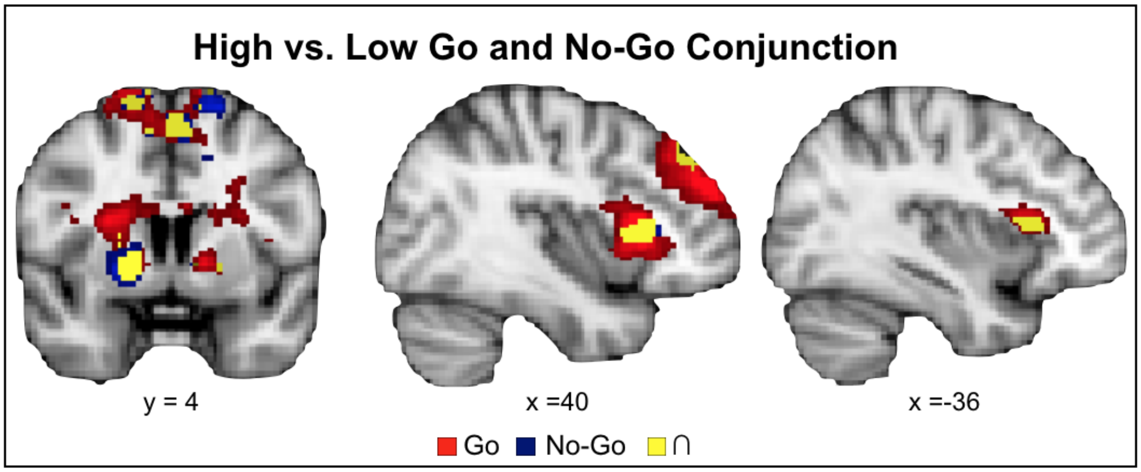

Supplementary Figure 3. High Stakes versus low stakes go and no-go conjunction. Whole brain corrected statistical maps illustrating regions exhibiting enhanced functional recruitment for high stakes relative to low stakes go trials (red) and no-go trials (blue). Overlap is shown in yellow. Contrast represents whole-brain corrected t-test thresholded at FWE $p<0.05$.

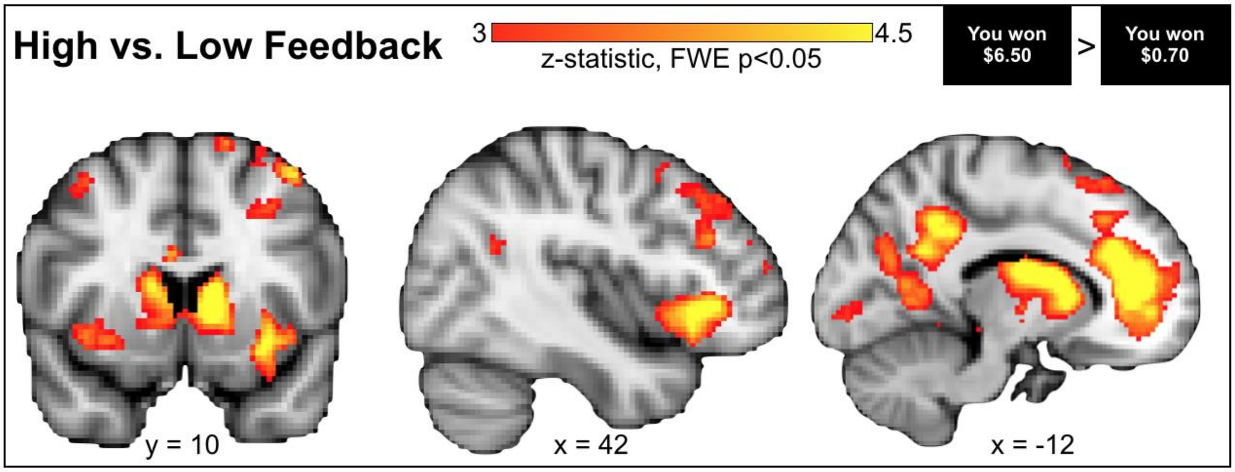

Supplementary Figure 4. High stakes versus low stakes feedback. Whole brain corrected statistical maps illustrating regions exhibiting enhanced functional recruitment for high stakes relative to low stakes feedback across all participants. Contrast represents whole-brain corrected t-test thresholded at FWE $p<0.05$.

Supplementary Table 1: Behavioral and MRI sample characteristics

	Behavioral Sample				MRI Sample			
**Age Group**	**N**	**M**	**F**	**Prop. M**	**N**	**M**	**F**	**Prop. M**
13-14	20	12	8	0.60	17	10	7	0.59
15-16	26	12	14	0.46	15	6	9	0.40
17-18	22	12	10	0.55	20	10	10	0.50
19-20	20	10	10	0.50	20	10	10	0.50
χ^2	χ^2=0.96, p=0.81				χ^2=1.13, p=0.77			
All	88	46	42	0.52	72	36	36	0.50

N: number of participants. M: male. F: male. Prop. M: proportion male.
χ^2: assessment of gender distribution across age

Supplementary Table 2. Mean estimated IQ by year of age

**Age**	**13**	**14**	**15**	**16**	**17**	**18**	**19**	**20**
**IQ**	103.0 (16.2)	103.7 (12.4)	110.2 (12.7)	103.7 (9.8)	113.1 (14.7)	110.4 (16.6)	103.1 (11.6)	111.7 (14.7)

Standard deviation is noted in parentheses.

Supplementary Table 3. Mean performance metrics by 2 year binned age groups

**Behavioral Measure**	**13-14**	**15-16**	**17-18**	**19-20**
Low Go RT (ms)	498.86 (75.43)	487.46 (53.18)	486.33 (81.57)	495.29 (114.99)
Low Go Acc (%)	96.41 (6.97)	98.01 (4.48)	96.73 (8.41)	98.36 (2.93)
Low No-go Acc (%)	74.63 (25.24)	79.07 (17.85)	85.12 (14.61)	85.47 (10.35)
Low dprime	2.94 (1.17)	2.84 (1.03)	3.35 (0.96)	3.39 (0.72)
High Go RT (ms)	496.60 (75.42)	479.67 (47.71)	489.92 (82.75)	489.33 (119.75)
High Go Acc (%)	96.80 (6.38)	97.42 (4.76)	97.39 (6.84)	99.30 (1.48)
High No-go Acc (%)	74.13 (20.22)	80.32 (14.54)	85.90 (12.87)	87.19 (11.64)
High dprime	2.84 (1.03)	3.07 (0.69)	3.38 (0.73)	3.62 (0.60)

Standard deviation is noted in parentheses. RT: reaction time. Acc (%): percent correct. Low: low stakes. High: high stakes.

Supplementary Table 4: Correlation matrix for performance measures

Correlation Matrix for Performance Measures					
	**Age**	**Go**	**No Go**	**RT**	**d'**
**Age**		0.13	0.28**	0.00	0.28**
**Go Accuracy**			0.10	-0.10	0.60***
**No-go Accuracy**				0.40***	0.81***
**RT**					0.26**
**d'**					

*p<0.01, **p<0.001, ***p<0.0001

Supplementary Table 5: FMRI data censoring correlations with age and performance

Data Censoring Not Related to Age or Performance		
	# volumes removed for motion (>1 mm)	total # volumes removed (motion & signal outliers)
**Age**	-0.06	0.04
**d'**	-0.12	-0.07
**$d'_{high-low}$**	-0.14	-0.11

Supplementary Table 6. High versus low stakes cue cluster table

High > Low Stakes Cue Contrast, whole-brain FWE p<0.05					
**Region**	**x**	**y**	**z**	**k**	**z-stat**
**Intracalcarine Cortex**	4	-86	4	20011	8.35
Occipital Pole	-8	-92	2		8.01
Intracalcarine Cortex	-16	-82	6		7.48
Lingual Gyrus	-8	-72	0		6.88
Pallidum	-16	-4	-6		6.14
Lingual Gyrus	20	-60	2		5.82
Caudate	-8	6	2		5.35
Putamen	-14	12	-8		5.32
Thalamus	-16	-16	12		5.29
Cuneal Cortex	2	-78	30		5.2
Lateral Occipital Cortex	-14	-82	38		5.2
Pallidum	16	-2	-6		5.02
Occipital Cortex	-26	-52	-22		4.99
Brainstem	6	-26	-4		4.92
Brainstem	-10	-26	-8		4.9
Occipital Cortex	-34	-50	-24		4.84
Lateral Occipital Cortex	44	-82	16		4.83
Insular Cortex	34	4	12		4.81
Thalamus	10	-20	0		4.7
Insular Cortex	-38	-2	-10		4.51
Occipital Fusiform Gyrus	-30	-78	-10		4.51
Parahippocampal Gyrus	26	-40	-2		4.22
Occipital Pole	14	-94	28		4.15
Parahippocampal Gyrus	-14	-38	-16		4.13
Putamen	24	2	2		4.08
Planum Polare	46	4	-10		3.96
Precuneuous Cortex	18	-72	38		3.85
**Precentral Gyrus**	-44	-10	52	9728	5.91
Superior Frontal Gyrus	-16	-8	66		5.62
Supplementary Motor Cortex	-10	6	46		5.57
Postcentral Gyrus	-46	-24	52		5.31
Precuneous Cortex	14	-38	46		5.31
Precentral Gyrus	44	-6	50		5.12
Cingulate Gyrus	4	-2	42		4.96
Superior Frontal Gyrus	10	-2	70		4.85
Supplementary Motor Cortex	10	2	54		4.79
Cingulate Gyrus	-8	-30	38		4.73
Superior Parietal Lobule	22	-46	50		4.57
Superior Parietal Lobule	-30	-52	56		4.55
Precuneous Cortex	-6	-46	52		4.48
Supramarginal Gyrus	-54	-44	30		4.05
Paracingulate Gyrus	-8	28	30		3.92
**Frontal Operculum Cortex**	-34	26	6	198	4.83
**Central Opercular Cortex**	-36	2	14	472	4.61
Frontal Operculum Cortex	-30	12	22		4.51
Insular Cortex	-32	10	10		4.37
Inferior Frontal Gyrus	-42	12	16		4.03
Middle Frontal Gyrus	-32	24	24		3.48
**Frontal Pole**	-32	40	32	269	4.15
Middle Frontal Gyrus	-26	30	30		4.05
Frontal Pole	-24	36	34		3.95
**Precentral Gyrus**	54	6	8	26	3.79
**Postcentral Gyrus**	44	-20	38	17	3.60
Supramarginal Gyrus	50	-24	36		3.42
**Supramarginal Gyrus**	66	-44	26	8	3.49
**Precentral Gyrus**	60	4	14	6	3.46

Supplementary Table 7. High versus low stakes feedback cluster table

High > Low Stakes Feedback Contrast, whole-brain FWE p<0.05					
**Region**	**x**	**y**	**z**	**k**	**z-stat**
**Insular Cortex**	-30	20	-8	1322	7.29
Frontal Orbital Cortex	-44	22	-14		4.85
Inferior Frontal Gyrus	-54	22	2		3.85
**Frontal Orbital Cortex**	28	20	-8	1449	6.61
Inferior Frontal Gyrus	54	26	18		3.70
**Paracingulate Gyrus**	-4	38	30	13623	6.57
Cingulate Gyrus	8	36	22		6.39
Paracingulate Gyrus	12	50	4		6.19
Cingulate Gyrus	-8	38	6		5.99
Precuneous Cortex	-4	-48	44		5.62
Frontal Pole	10	58	14		5.30
Frontal Pole	-24	38	42		5.19
Precuneous Cortex	12	-50	42		5.08
Middle Frontal Gyrus	-30	26	42		4.98
Superior Frontal Gyrus	-22	32	44		4.97
Superior Frontal Gyrus	8	42	44		4.84
Frontal Medial Cortex	-6	50	-10		4.82
Intracalcarine Cortex	14	-76	10		4.22
Supracalcarine Cortex	-8	-66	16		4.15
Middle Frontal Gyrus	38	26	46		4.11
Lingual Gyrus	-10	-60	4		3.98
Lingual Gyrus	0	-66	6		3.93
Intracalcarine Cortex	-16	-62	6		3.58
**Caudate**	-12	4	14	2516	5.93
Caudate	14	4	16		5.59
Thalamus	-6	-16	14		5.38
Thalamus	2	-10	4		5.09
**Middle Temporal Gyrus**	60	-48	10	714	4.99
Middle Temporal Gyrus	60	-48	10		4.99
Angular Gyrus	54	-44	24		4.80
Lateral Occipital Cortex	52	-60	22		4.27
**Angular Gyrus**	-54	-54	32	1171	4.87
Supramarginal Gyrus	-56	-50	48		4.58
Lateral Occipital Cortex	-46	-60	36		4.49
**Midbrain**	0	-26	-20	51	3.72
**Occipital Fusiform Gyrus**	-26	-74	-14	213	4.14
Occipital Cortex	-26	-54	-10		4.13
**Frontal Pole**	-26	46	-10	16	4.12
**Middle Temporal Gyrus**	62	-18	-6	116	3.97
**Occipital Pole**	-8	-90	-4	49	3.92
**Parahippocampal Gyrus**	24	-22	-6	32	3.88
**Superior Frontal Gyrus**	-16	10	68	11	3.84
**Precuneous Cortex**	22	-54	6	23	3.77
**Occipital Pole**	0	-94	12	27	3.71
**Middle Temporal Gyrus**	-50	-24	-8	22	3.70
**Frontal Pole**	40	56	14	16	3.64
**Lateral Occipital Cortex**	-48	-76	22	8	3.53

Supplementary Table 8: Structural ROI correlations with age and performance

Region	Analysis	Age	d’	$d'_{high-low}$
**Left Pars opercularis**	Thickness	-0.42**	-0.01	-0.03
	Volume	-0.33	-0.25	-0.04
Right Pars opercularis	Thickness	-0.43**	-0.14	-0.05
	Volume	-0.32	-0.09	-0.16
Left Pars triangularis	Thickness	-0.50***	-0.38*	0.10
	Volume	-0.07	0.06	-0.12
Right Pars triangularis	Thickness	-0.49***	-0.33	-0.00
	Volume	-0.08	-0.19	-0.11
Left Caudate	Volume	-0.09	-0.14	0.07
Right Caudate	Volume	-0.04	-0.09	0.02
Left Putamen	Volume	-0.16	-0.04	0.05
Right Putamen	Volume	-0.11	0.01	0.07
Left Accumbens	Volume	-0.06	0.21	-0.03
**Right Accumbens**	Volume	-0.22	-0.09	0.05

Multiple comparison threshold p=0.003; *p<0.003, **p<0.0003, ***p<0.00003
Bolded regions included as covariates in mediation analysis

Supplementary References

1. Fischl, B. FreeSurfer. *Neuroimage* **62,** 774–781 (2012).

2. Tamnes, C. K. *et al.* Development of the cerebral cortex across adolescence: a multisample study of inter-related longitudinal changes in cortical volume, surface area, and thickness. *J. Neurosci.* **37,** 3402–3412 (2017).

3. Desikan, R. S. *et al.* An automated labeling system for subdividing the human cerebral cortex on MRI scans into gyral based regions of interest. *Neuroimage* **31,** 968–980 (2006).
